# From variant of uncertain significance to likely pathogenic in two siblings with atypical RAG2 Deficiency: a case report and review of the literature

**DOI:** 10.1186/s12887-024-04597-2

**Published:** 2024-02-13

**Authors:** Nima Taghizadeh Mortezaei, Soha Mohammadi, Hassan Abolhassani, Sima Shokri, Mohammad Nabavi, Morteza Fallahpour, Mohammad Hassan Bemanian

**Affiliations:** 1 School of Medicine, Iran University of Medical Sciences (IUMS), Tehran, Iran; 2 Research Center for Immunodeficiencies, Children’s Medical Center, Tehran University of Medical Sciences, Tehran, Iran; 3 Division of Immunology, Department of Medical Biochemistry and Biophysics, Karolinska Institutet, Stockholm, Sweden; 4 Department of Allergy and Clinical Immunology, School of Medicine, Iran University of Medical Sciences (IUMS), Rasool-e-Akram Hospital, Mansoori ave, Sattarkhan street, Tehran, 14456 13131 Iran

**Keywords:** Inborn errors of immunity, Primary immunodeficiency, Recombination activating gene, Severe combined immunodeficiency, VUS classification, Likely pathogenic variant, Leaky SCID

## Abstract

**Background:**

Severe combined immunodeficiencies (SCIDs) are hereditary disorders characterized by impaired T and B cell function, resulting in significant immune system dysfunction. Recombination-activating gene (*RAG*) mutations account for a substantial proportion of SCID cases. Here, we present two sibling cases of SCID caused by a novel *RAG2* gene mutation.

**Case Presentation:**

The index case was an 8-year-old boy who had a history of recurring infections. After a comprehensive immunological workup, the initial diagnosis of agammaglobulinemia was revised to combined immunodeficiency (CID). The patient underwent hematopoietic stem cell transplantation (HSCT) but succumbed to cytomegalovirus (CMV) infection. His brother, a 4-month-old boy, presented with CMV chorioretinitis. Leaky SCID was diagnosed based on genetic tests and immunological findings. The patient received appropriate treatment and was considered for HSCT. Both siblings had a homozygous *RAG2* gene variant, with the first case classified as a variant of uncertain significance (VUS). The presence of the same mutation in the second brother, and the clinical phenotype, supports considering the mutation as likely pathogenic.

**Conclusions:**

This case report highlights a novel *RAG2* gene mutation associated with CID. The classification of a VUS may evolve with accumulating evidence, and additional studies are warranted to establish its pathogenicity. Proper communication between genetic counselors and immunologists, accurate documentation of patient information, increased public awareness, and precise utilization of genetic techniques are essential for optimal patient management.

## Background

Severe combined immunodeficiencies (SCIDs) are hereditary disorders that cause significant immune system dysfunctions. T and B cells, derived from the thymus gland and bone marrow, are absent or impaired due to these dysfunctions. As a result, humoral and cellular adaptive immunity are compromised [1]. SCID can be caused by seventeen molecular defects, with recombination-activating genes (*RAG1/2*) mutations accounting for almost half of T^−^ B^−^ SCID cases, depending on ethnicity [2, 3]. RAG1/2 proteins play a vital role in the VDJ (Variable, Diversity, and Joining) recombination process, which generates a diverse range of antigen receptors [4].

Patients with RAG-dependent immunodeficiency can present with various clinical phenotypes, ranging from classical T^−^ B^−^ SCID to Omenn Syndrome. However, a selected group of patients also exhibit atypical SCID or leaky SCID because their clinical characteristics do not precisely match the other two categories. T cells can be found in the peripheral blood of such patients but in limited numbers and dysfunctional mode [5, 6].

The American College of Medical Genetics and Genomics (ACMG) categorizes sequence variants as pathogenic, likely pathogenic, benign, likely benign, or of uncertain significance. A variant of uncertain significance (VUS) is a genetic variant identified through genetic testing but whose importance to human function or health is unknown. The term likely pathogenic is used to mean that a variant is disease-causing with greater than 90% certainty based on evidence about allele frequency in the population, computational prediction, functional assays, segregation, and de-novo data in the family [7]. In some specific occasions, if VUS mutation has been identified in multiple affected individuals in the same family or unrelated individuals with a similar phenotype, it suggests that it may be causative and could be labeled as likely pathogenic [7]. Our study reports two siblings with leaky SCID caused by the same mutation. The index case presents with a VUS mutation, and the same mutation in the second case confirmed the main genetic defect in the family.

## Case presentation

### Case 1

The proband was an 8-year-old boy born to second-degree consanguineous parents. He had a history of recurrent infections during infancy, including herpetic whitlow, pansinusitis, urticarial vasculitis, mastoiditis, purulent otitis, and recurrent pneumonia associated with peri-bronchial thickening in central areas of both lungs. There was a significant family history involving the deaths of three parental uncles of the patient without specific diagnoses at the ages of 3 months, six months, and five years. On physical examination, submandibular lymphadenopathy and a Bacillus Calmette-Guerin (BCG) vaccine scar were noted, which was injected at his birth based on national vaccination protocol. The patient did not exhibit any syndromic facial features or organomegaly, and the results of other clinical assessments were normal. Skin biopsy from the persisting skin lesions in the thighs, buttocks, hands and face were performed and indicated perivascular and perifollicular lymphohistiocytic infiltrate with vague granulomatous lesion.

Initially, based on the patient’s symptoms and the results of immunoglobulin and flow cytometry.

tests, which revealed a low serum total and the specific antibody, decreased isohemagglutinin titer, and severe reduction of CD20 + B cells; the patient was diagnosed with agammaglobulinemia. He was treated with intravenous immunoglobulin (IVIg), but not only did some symptoms persist, but new symptoms, such as muscle weakness, failure to thrive (FTT), and chronic bloody diarrhea also emerged. At first, these new findings were attributed to concomitant dermatomyositis and B cell deficiency; however, after considering the patient’s clinical course, consulting with several immunologists, and conducting an immunological workup (Table 1), the diagnosis of combined immunodeficiency (CID) was established. Serial immunologic tests of the patient revealed a progressive complex profile of inborn errors of immunity, which encompasses a slight decline in the count of white blood cells (WBC), accompanied by lymphopenia, below-average levels of CD3 and CD4 cells, and abnormalities in B cell indices. Molecular diagnosis was conducted using whole exome sequencing and analyzed based on a previously published method [8], and the results confirmed the presence of two novel VUS mutations in *RAG2* (homozygous c.1241G > T, p.G414V) and *BTK* (hemizygous c.1064 A > G, p.D355G) genes, both with low minor allele frequency in the population and predicted high damaging scores on the protein (Table 2).

**Table 1:** Immunological Workup of two cases with Combined Immunodeficiency

Parameters	Case1	Case2
WBC (*10^9^ / L)	4.86 (5.5–15.5)	3.68 (6.7–14.0)
Hb (g/L)	126 (115–155)	79 (95–135)
Plts (*10^9^ / L)	383 (150–450)	526 (150–450)
PMN (*10^9^ / L)	2.02 (1.5–8.5)	1.693 (1.00–8.00)
Eosinophils (*10^9^ / L)	0.25 (0.02–0.40)	-
Monocytes (*10^9^ / L)	0.68 (0.12–0.70)	-
Lymphocytes (*10^9^ / L)	1.992 (2.00–10.00)	1.324 (3.90–9.00)
CD3 (*10^9^ / L)	1.216 (1.40–3.70)	0.609 (2.50–5.60)
CD4 (*10^9^ / L)	0.338 (0.70–2.20)	0.106 (1.80-4.00)
CD8 (*10^9^ / L)	0.797 (0.490–1.30)	0.728 (0.59–1.60)
CD4/45RA (% lymphocyte)	45 (53–86)	8.2 (77–94)
CD4/45RO (% lymphocyte)	39 (9–26)	90.1 (3–16)
Naïve CD4 + T cells (% T4 cells)	2.1 (32–71)	-
Central memory CD4 + T cells (% T4 cells)	7.8 (10–39)	-
Effector memory CD4 + T cells (% T4 cells)	83.6 (9–39)	-
Naïve CD8 + T cells (% T8 cells)	3.8 (19–76)	-
Central memory CD8 + T cells (% T8 cells)	1.5 (0.3-9)	-
Effector memory CD8 + T cells (% T8 cells)	61.8 (6–33)	-
CFSE-division index	0.4 (> 0.1)	-
CFSE-proliferation index	1.0 (> 1.0)	-
CFSE-% of division cells	45.2 (> 12.0)	-
CD16/56 (*10^9^ / L)	0.338 (0.13–0.72)	0.08 (0.17–0.83)
CD19 (*10^9^ / L)	0.258 (0.39–1.40)	0.13 (0.43-3.00)
CD20 (*10^9^ / L)	0.048 (0.12–0.40)	-
Naïve B cells (% B cells)	90.5 (32–76)	-
Transitional B cells (% B cells)	1.6 (1.0–12.0)	-
Non-switched memory B cells (% B cells)	0 (1.2–10)	-
Switched memory B cells (% B cells)	3.9 (2–16)	-
CD21 low B cells (% B cells)	1.6 (0.1-4.0)	-
IgG (g/L)**	0.09 (3.86–14.70)	4.03 (0.49–6.11)
IgM (g/L)	0.10 (0.37–2.24)	1.40 (0.16–1.58)
IgA (g/L)	undetectable (0.25–1.54)	0.11 (0.07–0.89)
IgE (IU/mL)	2 (< 100)	-
Anti Tetanus (IU/ml)	0.01 (> 0.1)	-
Anti Diphtheria (IU/ml)	0.01 (> 0.1)	-
Isohemagglutinin titer	1/2 (< 1/8)	-
HIV	Negative	Negative
CMV PCR qualitative	-	Positive
CMV PCR quantitative (copies/mL)	-	13,591, 8,528
CMV IgM (U/mL)	-	4.1
CMV IgG (U/mL)	-	17.4
TREC	-	Undetected (≥ 5,862 per 10^6^ cells)
KREC	-	Undetected (≥ 14,382 per 10^6^ cells)
AST (U/l)	47 (< 37)	59 (< 37)
ALT (U/l)	62 (< 41)	41 (< 41)
ALP (U/l)	236 (180–1200)	-

Note: The units are presented in International System of Units (SI units). All normal ranges cited here are adapted to the age [22, 23]. *Abbreviations: WBC: White Blood Cells; Hb: Hemoglobin; Plts: Platelets; PMN: Polymorphonuclear Cells; CD: Cluster of Differentiation; CFSE: Carboxy fluorescein Succinimidyl Ester; Ig: Immunoglobulin; HIV: Human Immunodeficiency Virus; CMV PCR: Cytomegalovirus Polymerase Chain Reaction; TREC: T-cell Receptor Excision Circles; KREC: Kappa-deleting Recombination Excision Circles; AST: Aspartate Aminotransferase; ALT: Alanine Aminotransferase; ALP: Alkaline Phosphatase*

** Before initiation of immunoglobulin replacement therapy

**Table 2:** Novel genetic variants in known inborn errors of immunity genes identified in two siblings with atypical SCID

Parameters	Gene1	Gene2
Gene	*RAG2*	*BTK*
Inheritance	AR	XLR
Chromosome position	Chr11: 36,614,478	ChrX:100,609,657
Variant location	Exon 2	Exon 14
DNA change	c.G1241T	c.A1064G
Protein change	p.G414V	p.D355G
Zygosity	Homozygous	Hemizygous
Classification	VUS	VUS
GnomAD population frequency	0	0
CADD score	27.0	22.5
ACMG criteria	PM2 PP3	PM2 PP5 BP4
ACMG criteria in two siblings	PP1	-
Reported in case 1	+	+
Reported in case 2	+	-

*Abbreviations: VUS: Variant of Uncertain Significance, AR: Autosomal Recessive, XLR: X-linked Recessive*

The segregation analysis of the parents unveiled that both parents carried the heterozygous *RAG2* variant. Additionally, the mother exhibited an additional genetic characteristic; she was found to be a carrier of the *BTK* variant. Taken together, these findings indicated the compromised development of both B and T cells, was likely attributed to the *RAG2* mutation. Furthermore, early reduced levels of CD19 and CD20 compared to T cells, along with decreased levels of immunoglobulins, underscore a significant deficiency in B cells, which can be assigned to the presence of the *BTK* mutation. Moreover, the progressive unbalanced distribution of cellular immunity observed in the patient, potentially influenced by the effects of both mutations, as recent study indicated the impaired cellular immunity in BTK deficiency, including abnormal memory T cells, T regulatory cells, delayed cell-mediated immune responses, defective T helper cell polarization and impaired T cell receptor diversity [9].

Meanwhile the patient was candidate for hematopoietic stem cell transplantation (HSCT), he received prophylactic therapy with antiviral, antifungal, and antibiotic medications, and the administration of IVIg was maintained. Consequently, in June 2020, he underwent HSCT with his mother as the donor; however, unfortunately, 1-month post-transplantation, the patient became infected with cytomegalovirus (CMV) and passed away at 8 years.

### Case 2

The second patient was the sibling of case 1, and since the patient had a family history of atypical SCID in his brother, he was undergoing a prenatal diagnostic amniocentesis test performed before birth, which revealed the same VUS *RAG2* variant as his brother, but without the *BTK* variant (Table 2). In spite of genetic consultation, the parents decide on the continuation of the pregnancy. He was admitted to the neonatal intensive care unit (NICU) due to being born prematurely at 31 weeks of gestational age. Despite being aware of their child’s condition, the parents chose to disregard it, leading to the administration of the BCG vaccine when the child was two months old.

The patient did not present any infection or inborn errors of immunity symptoms until four months of age, after which he was referred to our hospital for retinopathy of prematurity (ROP) screening. During the patient’s ROP screening, hemorrhagic lesions with a white granular appearance were observed on the retina, with the highest activity at the margins indicating CMV chorioretinitis. However, there was no evidence of CMV involvement in the central nervous system (CNS) as the neurological examinations, brain computed tomography (CT) scan, and lumbar puncture (LP) results were normal.

Due to an opportunistic infection and the history of his brother, the patient underwent a full immunological workup (Table 1). The findings indicate that the patient exhibited leukopenia, lymphopenia, and reduced numbers of CD3, CD4, CD16/56, and CD19 cells, indicating impaired T-cell development and compromised cellular immunity. Additionally, the disproportionate distribution of CD4/45RA and CD4/45RO percentages further emphasizes the disruption in T cell populations. Collectively, these observations align with the presence of the *RAG2* mutation in the patient. Consequently, based on the results of genetic tests and flow cytometry, a diagnosis of leaky SCID was established. Additional examinations, such as the T-cell receptor excision circle (TREC) and kappa-deleting recombination excision circle (KREC) evaluations, were carried out. The TREC analysis functions as an indicator of the existence of recent thymic emigrants, while the KREC assessment is suggestive of B-cell development. In this particular instance, both the TREC and KREC assessments yielded undetectable results, solidifying SCID diagnosis. Despite the low level of CD19 + B cells, the second case had normal serum IgM levels and also more prominent production of switched immunoglobulins, IgG and IgA, emphasizing the possibility of pathogenicity of BTK variant in the proband. The patient was admitted and received intravenous ganciclovir treatment for CMV infection. Additionally, he was taking antituberculosis medication (rifampin, isoniazid, and ethambutol) due to receiving the BCG vaccine and antifungal medicine (fluconazole). He was also given trimethoprim-sulfamethoxazole (TMP-SMX) as prophylaxis and IVIg. The patient was evaluated by the transplant committee and considered a candidate for HSCT and is on the waiting list.

## Discussion and conclusions

**Fig. 1:**
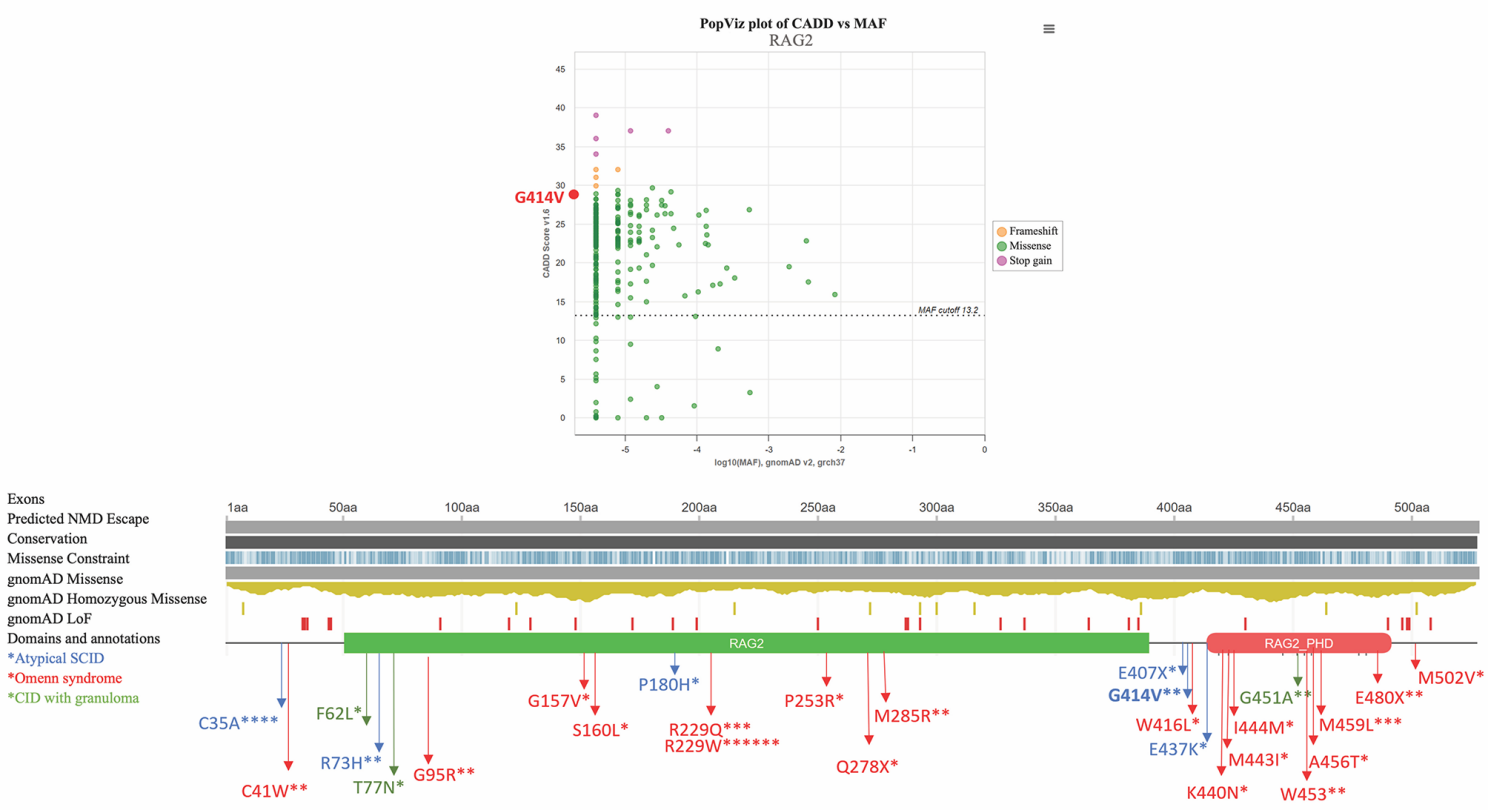
Comparing novel RAG2 p.G414V mutations in atypical SCID to reported gene hypomorphisms

**Fig. 2:**
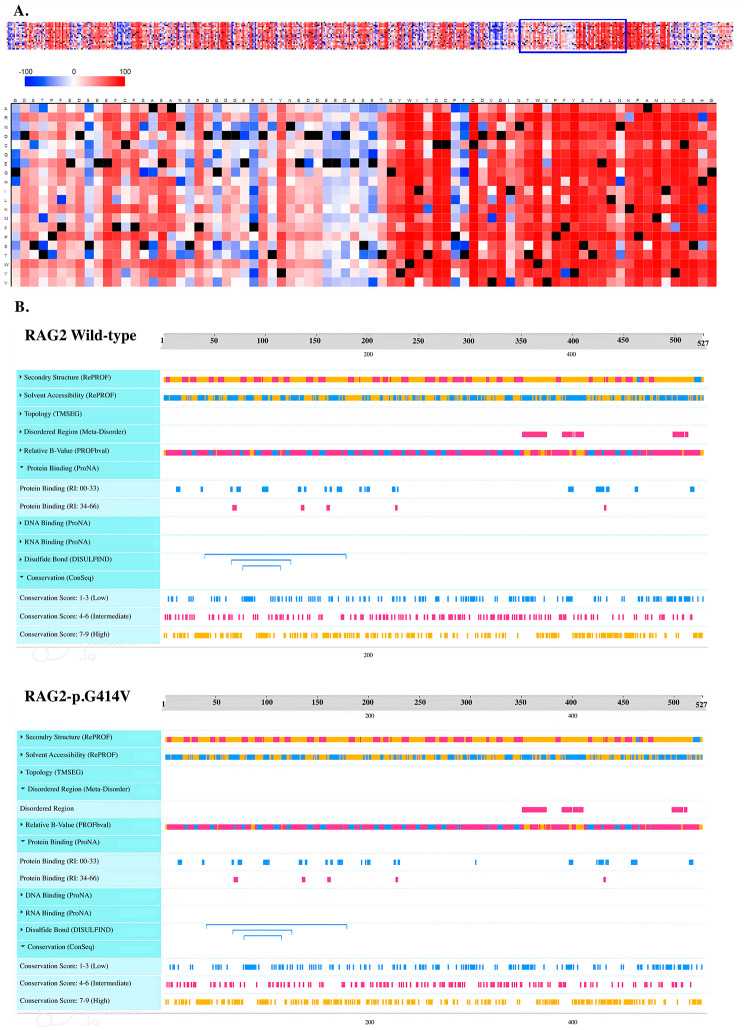
Assessing Structural Implications of RAG2 Mutations Using SNAP2 and Meta-Disorder Predictions. Structural effects of mutations predicted with SNAP2; a trained classifier that is based on a machine learning device called “neural network”. It distinguishes between effect and neutral variants/non-synonymous SNPs by taking a variety of sequence and variant features into account. The most important input signal for the prediction is the evolutionary information taken from an automatically generated multiple sequence alignment. Also, structural features such as predicted secondary structure and solvent accessibility are considered. The figure clearly showed the impact of mutation in the Glycine 414 and forward in the Zinc domain of the RAG2 mutations (**A**). Moreover, Meta-disorder predicted a disordered region and protein binging as the consequence of G414V mutation (**B**)

Our two sibling patients were found to carry a homozygous variant in the *RAG2* gene (c.1241G > T, p.G414V), which is considered a new mutation that could cause atypical SCID (Fig. 1). The first brother’s mutation was classified as a VUS. Over time, it is possible that the classification of a VUS may change, either by being reclassified as likely benign or benign, or towards a likely pathogenic or pathogenic classification. If a VUS mutation satisfies certain criteria, such as being observed in other individuals with the same phenotype indicating a potential link with a particular disorder, affecting the activity or expression of the gene or its protein product in vitro or in vivo functional studies, or being identified in multiple affected individuals in the same family or unrelated individuals with a similar phenotype, it may be considered likely pathogenic [7, 10]. This variant has not been described in the Exome Aggregation Consortium, Exome Sequencing Project, or the 1000 Genomes Browser to date. The structural analysis of the protein showed that sine glycine (G) and valine (V) are both non-polar amino acids with aliphatic R groups, but glycine has no side chain whereas valine is bulkier due to its side chain. A change from glycine to valine can thus potentially disrupt the local folding of the protein in the D/E-rich domain in the zinc finger of the RAG2 protein. Moreover, protein binding of the plant homology domain (PHD) might specifically impact the binding to the trimethylated lysine residue of histone H3K4me3 (Fig. 2). Given the presence of the same mutation in the second brother with a similar phenotype to his sibling and the absence of other *BTK* mutations, the variant can be considered likely pathogenic.

Benhsaien et al. identified a new *RAG2* mutation (c.826G > A) in two cases with a similar clinical phenotype. While the variant was classified as a VUS, the authors concluded that it is likely responsible for the SCID based on the observed clinical phenotype and familial segregation analysis, specifically, the presence of the same variants in the heterozygous state in un-affected family members [11]. The study by Alizadeh et al. examines SCID and atypical SCID cases, including eight male and female patients who exhibited SCID symptoms early in infancy. Two patients presented with novel mutations in the *RAG2* gene; one is a missense mutation (c.818T > C) and the other is a stop-gained mutation (c.130G > T), leading to atypical and typical SCID, respectively. These mutations underscore the pivotal role of RAG2 in immune system processes, including B cell differentiation and T cell development in the thymus, emphasizing the importance of understanding these genetic variations in the context of SCID [12]. . Karaatmaca et al. conducted a 20-year study on RAG 1/2 deficient patients, identifying nine new mutations among 35 patients (25 RAG1 deficient, 10 RAG2 deficient) including c.217 C > T/ c.712delG, c.707T > G, c.1886 C > T, c.1782 C > A, c.951G > T, c.746 G > A, c.712delG, c.1280_1281insTGGATAT. Twenty-five patients were diagnosed with typical T-B-NK + SCID, while others had OS and delayed-onset CID. Some patients experienced CMV infection, with two SCID patients developing retinitis. Out of these patients, 28 underwent HSCT, while seven patients did not survive to undergo HSCT [13]. Chitty-Lopez et al. demonstrate the significance of the T-cell receptor excision circle (TREC) assay in newborn screening for detecting T-cell lymphopenia (TCL) and identifying SCID. They present a case of an asymptomatic newborn with undetectable TRECs, revealing severe TCL but normal B cell quantities and lymphocyte proliferation. Next-generation sequencing identified compound heterozygous hypomorphic RAG variants, including one novel. The patient received successful HSCT treatment. This case highlights the importance of in vitro studies to confirm variant pathogenicity and expedite definitive treatment, reducing the risk of complications [14]. Korkmaz et al. reviewed 59 patients diagnosed with SCID over 20 years to develop a diagnostic algorithm for countries with high consanguineous marriage rates that lack newborn TREC assay screening. The key findings include frequent symptoms of cough, eczematous rash, and organomegaly. The common genetic defects identified were ADA, Artemis, RAG1/2, MHC Class II, and IL-2R. Most patients had lymphocyte counts below 3000/mm3. The study suggests that, for early diagnosis in countries with high consanguinity rates, emphasis should be on children under two years exhibiting severe infections, rashes, and low lymphocyte counts [15].

The diagnosis of patient 1 poses a tough challenge, primarily due to the simultaneous presence of two distinct primary immunodeficiency disorders. The initial disorder is X-linked agammaglobulinemia (XLA), resulting from a mutation in the *BTK* gene, and the second disorder is a suboptimal response CID, originating from a mutation in the *RAG2* gene. What complicates this diagnosis is that the patient’s clinical symptoms did not neatly correspond to the distinctive features of either condition but instead displayed a perplexing combination of characteristics from both diseases. This scenario highlights the significance of contemplating alternative explanations when a solitary disorder fails to explain the symptoms’ manifestation adequately. In a study by Estébanez et al., a 2-year-old boy exhibited puzzling symptoms, including severe skin lesions. Genetic analysis uncovered a heterozygous *RAG2* mutation (c.644 C > T). Unlike classical severe immune disorders linked to *RAG2* mutations, this patient exhibited a distinct clinical profile, marked by eczematous/ichthyosiform skin issues, growth impairment, lymphadenopathy, alopecia, eosinophilia, and elevated serum IgE levels. Notably, the patient’s mother carried the same mutation but remained asymptomatic. Over three years, the patient’s skin condition improved, though elevated eosinophil counts and IgE levels persisted, necessitating ongoing observation. This study reveals a novel clinical manifestation associated with a heterozygous *RAG2* mutation, emphasizing the significance of advanced genetic diagnostics in understanding immunodeficiencies [16]. Lugo-Reyes et al. examined the impact of *RAG1/2* gene mutations on SCID by studying six patients from unrelated families with missense *RAG1* or *RAG2* variants. Two patients with the same *RAG2* variant (c.685 C > T) displayed different clinical outcomes; both had respiratory issues, but only one showed autoimmune manifestations. These variations may result from the specific mutation locations and their impact on recombinase V(D)J activity. This study emphasizes the varied effects of *RAG1/2* variants and the need to explore T lymphocyte diversity to understand RAG deficiency better [17]. Ozturk et al. studied 54 SCID patients, including classical SCID, OS, and atypical SCID, to assess outcomes and factors influencing prognosis post-HSCT. Most patients had the T-B-NK + phenotype with predominant RAG1/2 mutations. The post-HSCT survival rate was 83.3%. Factors enhancing recovery included peripheral blood stem cell sources, genotypes other than RAG, gender-matched transplants, and conditioning regimens. Active infections and mismatched donors reduced survival rates. The study emphasizes the importance of early diagnosis and tailored interventions for improved post-transplant outcomes [18]. Villa et al. explore genetic defects in recombination activating genes *RAG1* and *RAG2*, leading to a spectrum of severe immune issues. They highlight the need to develop novel therapeutic approaches, such as minimal conditioning regimens with monoclonal antibodies and gene therapy, to treat all RAG deficient patients, regardless of disease severity or donor availability. Researchers are working on strategies to give gene-corrected cells an advantage in overcoming differentiation blocks. Autologous gene-corrected stem cell transplants and genome editing at specific loci are emerging as promising techniques for treatment. These advancements represent a significant step forward in addressing RAG deficiencies [19].

To determine the pathogenicity of a VUS associated with SCID, it might be essential to collect more data. Additionally, genes not previously connected to SCID could be discovered. To further investigate, research methods such as the Matchmaker approach can be employed to find similar patients through the Clinical Immunology Society (Matchmakerexchange.org). Additionally, checking for other variants at the same amino acid on gnomAD (Gnomad.broadinstitute.org) and looking up the variant in the Catalogue of Somatic Mutations in Cancer (COSMIC) (Cancer.sanger.ac.uk/cosmic) may also provide helpful information [20].

Prenatal diagnosis can precisely identify fetal conditions, helping to prevent birth defects and reduce anxiety for expectant mothers. In recent years, gene diagnosis for patients’ family pedigrees using DNA samples has made significant progress, especially with high-throughput sequencing technology [21]. The usage and interpretation of genetic testing results depend on several factors, including the clinical context. In cases where prenatal testing is used, and a family must make crucial decisions such as fetal treatment or termination of pregnancy, it is essential to carefully evaluate all available information, including the test report and other sources, such as fetal ultrasound and previous genetic results before deciding on a course of action and here, the VUS variants can challenge the final decision for the family. In a prenatal setting, it is crucial to give families a clear explanation of the terminology of genetic variants whenever a genetic test outcome is the only accessible proof. Thus, referring healthcare professionals must interact with the clinical laboratory to comprehend how variations are categorized to give suitable patient counseling [7].

Considering the clinical course of patients, we suggest several recommendations to improve patient management, particularly for those with immunodeficiency. Firstly, establishing direct communication between genetic counselors and immunologists is recommended to prevent any missed diagnoses. Secondly, it is important to systematically record patient information at all levels of medical services to ensure that patients with primary immunodeficiency who are not eligible to receive live vaccines are not mistakenly vaccinated under general vaccination protocols and also the possibility of having more than one primary immunodeficiency in a single patient. Thirdly, increasing public awareness about primary immunodeficiency diseases is crucial. Lastly, for a better understanding and classification of variants, the precise and appropriate use of novel genetic techniques should be encouraged.

## Data Availability

All data generated or analyzed during this study are included in this published article.

## Abbreviations

SCIDs: Severe Combined Immunodeficiencies
RAG: Recombination-Activating Gene
VUS: Variant of Uncertain Significance
CMV: Cytomegalovirus
HSCT: Hematopoietic Stem Cell Transplantation
CID: Combined Immunodeficiency
IVIg: Intravenous Immunoglobulin
